# Editorial: Innovative approaches in precision radiation oncology

**DOI:** 10.3389/fmed.2026.1776567

**Published:** 2026-01-22

**Authors:** Abdul K. Parchur, Poonam Yadav

**Affiliations:** 1 Department of Radiation Oncology, University of Maryland School of Medicine, Baltimore, MD, United States; 2 Department of Radiation Oncology, Feinberg School of Medicine, Northwestern University, Chicago, IL, United States

**Keywords:** adaptive radiotherapy, dosimetry and quality assurance, flash, imaging and verification, immuno-radiotherapy, precision radiation oncology, radiobiological modulation, treatment planning and delivery

Radiation therapy remains a cornerstone of cancer care, with an estimated ~50% of patients receiving radiotherapy at some point during their illness. Over the past decade, progress in image guidance, treatment delivery, computational modeling, and radiobiology has accelerated the shift toward precision radiation oncology, where treatment is increasingly adaptive, personalized, and data-driven. This Research Topic, *Innovative approaches in precision radiation oncology*, was developed to highlight multidisciplinary innovations that improve precision across the radiotherapy pipeline, from imaging and planning to delivery, biology, and clinical implementation. Following rigorous peer review, ~50% of submitted manuscripts were accepted, resulting in a curated Research Topic of 25 articles authored by 212 contributors.

## Treatment planning, delivery, and optimization

A substantial portion of the Research Topic focused on innovations in treatment planning, delivery techniques, and optimization strategies aimed at improving dose conformity, organ-at-risk (OAR) sparing, and clinical feasibility across diverse disease sites. Several studies addressed emerging paradigms for spatial dose modulation and high-precision delivery in challenging clinical scenarios.

Advancing spatially fractionated radiotherapy, Ma et al. introduced an optimization framework for determining vertex placement in lattice radiotherapy using closest packing combined with adaptive simulated annealing. Their method significantly increased the peak-to-valley dose contrast, with the optimized plans demonstrating a nearly six-fold improvement in peak-to-valley index compared with conventional packing approaches, highlighting a reproducible and automated strategy to enhance treatment precision for bulky tumors. Complementing this work on spatial dose modulation, Li et al. evaluated proton-based stereotactic centralized/core ablative radiation therapy (pSCART) in a treatment planning study of bulky tumors. By leveraging the physical dose advantages of protons, pSCART enabled higher prescription doses (24 Gy × 3 fractions) and improved high-dose central coverage while reducing dose spill at the tumor periphery and lowering mean doses to surrounding OARs compared with photon-based SCART techniques, supporting its potential for future clinical translation.

Also, contributions focused on optimization of volumetric modulated arc therapy (VMAT) planning parameters to improve OAR protection without compromising target coverage. Guo et al. demonstrated that careful adjustment of physical parameters in a knowledge-based partial-arc VMAT RapidPlan model for left-sided breast cancer significantly reduced doses to the heart, lungs, and contralateral breast, emphasizing the importance of aligning model configuration with delivery geometry to mitigate low-dose exposure and secondary cancer risk. In a related dosimetric study, Huang et al. showed that incorporating orthogonal collimator angles (0°/90°) in dual-arc VMAT improved conformity, dose gradient, and OAR sparing across head-and-neck, thoracic, and pelvic sites, with all plans maintaining high delivery accuracy, demonstrating a simple yet effective planning refinement applicable in routine practice. Optimization of biological and clinical dose constraints was further explored by Hu et al., who investigated the use of the Monaco serial biological function for cardiac dose optimization in deep-inspiration breath-hold IMRT for left-sided breast cancer. Their analysis identified an optimal K-value range (2–4) that achieved balanced reduction in mean and maximum cardiac and coronary artery doses while preserving target coverage, providing evidence-based guidance for biologically informed planning parameter selection. Innovations in system-specific planning were also highlighted. Tang et al. developed a hippocampal-sparing whole-brain radiotherapy planning approach using the Halcyon platform with coplanar dual-arc VMAT and target substructure segmentation. Compared with conventional systems, this strategy achieved improved dose homogeneity, reduced hippocampal dose and normal tissue complication probability, and high gamma passing rates, demonstrating that advanced planning techniques can enhance neurocognitive preservation without increasing workflow complexity. Furthermore, clinical feasibility of advanced planning and delivery workflows was reinforced by Sun et al., who evaluated fan-beam CT–guided online adaptive re-planning in definitive cervical cancer radiotherapy. Across 278 adaptive fractions, their approach consistently improved target coverage and OAR sparing with a mean gamma passing rate exceeding 99% and a clinically acceptable workflow duration of approximately 23 min, underscoring how optimized planning and delivery strategies can be successfully integrated into routine clinical practice. In the context of rare pediatric tumors, Zhang et al. reported a multimodal precision treatment strategy for MN1-altered astroblastoma, demonstrating how the tissue-sparing advantages of proton beam therapy can facilitate effective salvage Gamma Knife radiosurgery while maintaining durable local control and minimal toxicity.

## Imaging, dosimetry, and verification

Advanced imaging, quantitative dosimetry, and rigorous verification are foundational to precision radiation oncology, enabling accurate target definition, reliable dose delivery, and safe clinical implementation of increasingly complex treatment techniques. Six contributions in this Research Topic addressed these themes across clinical, technical, and preclinical domains, highlighting how imaging and verification strategies directly influence treatment accuracy, toxicity, and translational reliability.

Several studies focused on dosimetric characterization and verification of complex delivery techniques. Wang et al. performed a comprehensive dosimetric and efficiency comparison between TomoDirect and TomoHelical radiotherapy for total skin irradiation. Their analysis demonstrated that TomoDirect plans using more than nine beams achieved target coverage, homogeneity, and organ-at-risk sparing comparable to TomoHelical delivery, while maintaining similar treatment times when appropriate auxiliary structure blocking was applied. These findings provide practical guidance for modality selection and protocol design in total skin irradiation, where large treatment volumes and delivery efficiency are critical. Quantitative assessment of dose delivery accuracy and verification was further advanced by Zhou et al., who developed a lightweight Swin-Transformer–based deep learning framework for patient-specific VMAT delivered-dose prediction. Their model achieved high structural similarity and gamma passing rates relative to ground truth dose distributions, while substantially reducing model complexity compared with existing transformer architectures. This work highlights the growing role of artificial intelligence in patient-specific quality assurance, offering a scalable and clinically viable alternative to labor-intensive measurement-based verification workflows. The role of imaging in refining dosimetric evaluation and treatment response assessment was addressed in multiple contributions. Qian et al. analyzed quantitative imaging features derived from ^18^F-DOPA PET in glioblastoma patients treated with dose-escalated radiotherapy. Their results demonstrated that pre-treatment imaging signatures and early post-treatment changes could stratify survival outcomes in MGMT-unmethylated tumors and identify patients most likely to benefit from dose escalation. This study underscores the potential of functional imaging biomarkers to inform both dosimetric decision-making and adaptive treatment strategies. Similarly, Borowiec et al. evaluated the clinical value of extended baseline cross-sectional imaging in locally advanced high-risk breast cancer. By incorporating CT or PET-CT into initial staging, the authors showed that disease stage and radiation treatment planning were altered in a substantial proportion of patients, enabling highly customized dose delivery to surgically inaccessible nodal regions. Their findings emphasize that imaging choice at baseline can directly impact dosimetric strategy and treatment individualization. Geometric sensitivity and setup-related dose variation were examined by Xiao et al., who conducted a propensity-matched cohort study comparing supine and prone positioning in postoperative cervical cancer radiotherapy. While target coverage remained comparable, positioning significantly influenced organ dose distributions, setup error profiles, and acute toxicity patterns. This work highlights how verification of geometric and positional factors is integral to optimizing both dosimetry and clinical safety in pelvic radiotherapy. Finally, Tavakkoli et al. addressed verification and biological confounding factors in the preclinical evaluation of ultra-high dose-rate (FLASH) radiotherapy. Using a murine total abdominal irradiation model, they demonstrated that anesthetic-dependent tissue oxygenation critically determined the presence or absence of FLASH sparing. Their findings underscore the importance of rigorous control and reporting of physiological variables when verifying dose-response relationships, particularly for emerging technologies poised for clinical translation.

Collectively, these studies demonstrate that precision radiation oncology relies not only on sophisticated planning and delivery techniques but also on robust imaging, dosimetric evaluation, and verification frameworks. By integrating quantitative imaging, advanced computational verification, and careful assessment of geometric and biological uncertainties, these contributions provide essential foundations for safe, accurate, and personalized radiotherapy.

## Adaptive and online radiotherapy and clinical implementation

The transition from advanced treatment concepts to routine clinical practice represents a critical step in precision radiation oncology. Three contributions within this Research Topic focused on adaptive and online radiotherapy workflows, emphasizing real-time plan adaptation, clinical feasibility, and practical considerations for implementation in busy treatment environments.

Anchoring this theme, the technical feasibility of optimized planning and delivery was supported by Sun et al., who demonstrated high-accuracy fan-beam CT-guided online adaptive replanning in definitive cervical cancer radiotherapy, achieving consistent target coverage and organ-at-risk sparing across 278 fractions within a clinically acceptable workflow time. Building on this foundation, Asher et al. examined broader aspects of clinical implementation, focusing on workflow translation and operational considerations associated with adaptive radiotherapy adoption. Their study addressed practical challenges such as staffing, training, and integration of adaptive processes within existing clinical infrastructures, highlighting that successful implementation depends not only on technical capability but also on multidisciplinary coordination and institutional readiness. Complementing these perspectives, Pang et al. explored clinical adaptation strategies aimed at tailoring treatment delivery to patient-specific and time-varying anatomical changes. Their work emphasized decision-making frameworks for when and how to adapt treatment plans, underscoring the importance of balancing dosimetric benefit with workflow efficiency and clinical resource utilization.

Collectively, these studies demonstrate that adaptive radiotherapy has progressed beyond proof-of-concept, with emerging evidence supporting its safe and effective deployment in clinical practice (Yan et al.). By addressing real-time adaptation, workflow feasibility, and implementation strategy, these contributions provide a roadmap for translating adaptive radiotherapy technologies into routine patient care and advancing the clinical impact of precision radiation oncology.

## Biological determinants of radiation response and therapeutic modulation

A defining challenge in precision radiation oncology is understanding how identical physical dose prescriptions can produce markedly different biological and clinical outcomes across patients. Several contributions in this Research Topic addressed this challenge by examining immune signaling pathways, tumor-microenvironment interactions, and biological predictors of treatment response, highlighting the importance of integrating molecular biology into radiation treatment paradigms.

Xiong et al. provided a comprehensive mini-review of the cGAS-STING signaling pathway as a central mediator linking radiation-induced DNA damage to innate and adaptive immune activation in breast cancer. The authors emphasized the dual and subtype-dependent roles of cGAS-STING signaling, demonstrating that acute pathway activation can enhance antitumor immunity and radiosensitization, whereas chronic or dysregulated activation may promote immunosuppression, regulatory T-cell expansion, and therapeutic resistance. By integrating molecular mechanisms with emerging translational and clinical considerations across luminal, HER2-positive, and triple-negative breast cancer subtypes, this work underscores the need for subtype-specific immune modulation strategies when designing radiation-immunotherapy combinations.

Complementing immune-centric mechanisms, Keepers et al. investigated biological response modulation using mouse pancreatic tumor organoids as a translational preclinical platform. Their study demonstrated that combining low-dose chemotherapy with fractionated radiation produced significantly greater tumor growth inhibition than either modality alone. This enhanced effect was associated with increased DNA damage signaling, elevated reactive oxygen species production, and suppression of mesenchymal markers, supporting the concept that biologically informed combination strategies can amplify radiation efficacy while potentially reducing treatment-related toxicity. The use of tumor-derived organoids further highlights the value of advanced biological models for evaluating radiation–drug interactions and guiding translational research.

Biological heterogeneity and target expression were further examined by Peslier et al. in a clinical study of patients with metastatic castration-resistant prostate cancer treated with ^177^Lu-PSMA radioligand therapy. The authors identified clinical, biochemical, and PSMA PET-derived imaging factors associated with early treatment discontinuation and poor response, including markers of disease burden and reduced target expression. These findings emphasize that biological determinants of response play a critical role in radiopharmaceutical-based radiation treatments and reinforce the importance of biomarker-driven patient selection.

Finally, Zajac-Grabiec et al. reviewed non-cancer late effects following proton beam therapy in pediatric patients, focusing on neurocognitive, endocrine, cardiovascular, and brainstem toxicities. While proton therapy offers superior normal-tissue sparing compared with photon-based approaches, the review highlights that biological sensitivity of developing tissues remains a key determinant of long-term outcomes. This contribution underscores that precision radiation oncology must balance tumor control with age-dependent biological vulnerability when optimizing treatment strategies in pediatric populations.

These studies illustrate that precision radiation oncology extends well beyond technical optimization of dose delivery. Immune signaling pathways, tumor microenvironment biology, radiopharmaceutical target expression, and normal-tissue susceptibility all shape therapeutic response and toxicity. Integrating biological stratification, mechanistic insight, and biomarker-guided decision-making into radiation oncology practice will be essential for overcoming resistance, improving combination strategies, and advancing truly personalized radiation treatment.

Together, the contributions in this Research Topic demonstrate that precision radiation oncology extends beyond technical optimization of dose delivery to encompass imaging, adaptive workflows, biological modulation, and clinical implementation. By integrating advanced planning and verification, real-time adaptation, molecular and immune insights, and biomarker-guided decision-making, these studies collectively advance more effective, resilient, and truly personalized radiation treatment strategies.

